# Interferon gamma-induced protein 10 is associated with insulin resistance and incident diabetes in patients with nonalcoholic fatty liver disease

**DOI:** 10.1038/srep10096

**Published:** 2015-05-11

**Authors:** Chia-Chu Chang, Chia-Lin Wu, Wei-Wen Su, Kai-Lun Shih, Der-Cherng Tarng, Chen-Te Chou, Ting-Yu Chen, Chew-Teng Kor, Hung-Ming Wu

**Affiliations:** 1 Division of Nephrology, Department of Internal Medicine, Changhua Christian Hospital, Changhua, Taiwan; 2 School of Medicine, Chung-Shan Medical University, Taichung, Taiwan; 3 Institute of Clinical Medicine, National Yang-Ming University, Taipei, Taiwan; 4 Department of Gastroenterology, Changhua Christian Hospital, Changhua, Taiwan; 5 Division of Nephrology, Department of Medicine, Taipei Veterans General Hospital, Taipei, Taiwan; 6 Department and Institute of Physiology, National Yang-Ming University, Taipei, Taiwan; 7 Department of Medical Imaging, Changhua Christian Hospital, Changhua, Taiwan; 8 Inflammation Research & Drug Development Center, Changhua Christian Hospital, Changhua, Taiwan; 9 Internal Medicine Research Center, Changhua Christian Hospital, Changhua, Taiwan; 10 Graduate Institute of Acupuncture Science, China Medical University, Taichung, Taiwan; 11 Department of Neurology, Changhua Christian Hospital, Changhua, Taiwan

## Abstract

Nonalcoholic fatty liver disease (NAFLD) is an important risk factor for the development of type 2 diabetes mellitus. Interferon gamma-induced protein 10 (IP-10), a proinflammatory chemokine, plays a crucial role in inflammatory diseases. This cross-sectional pilot study investigated whether circulating IP-10 is associated with the progression of liver disease, and prediabetes in patients with NAFLD. A total of 90 patients with NAFLD alone (n = 48) or NAFLD with incident diabetes (n = 42) and 43 controls participated in this study. Fasting plasma was used to assess metabolic parameters, inflammatory factors, endotoxin levels, and malondialdehyde (MDA) concentrations. Insulin resistance was estimated using homeostatic model assessment (HOMA-IR). IP-10 levels were significantly higher in patients with NAFLD alone (median (interquartile range): 369.44 (309.30–418.97) pg/mL) and in those with incident diabetes (418.99 (330.73–526.04) pg/mL) than in controls (293.37 (214.10–331.57) pg/mL) (*P *< 0.001). IP-10 levels were positively correlated with levels of alanine aminotransferase, hs-CRP, MDA, MCP-1, and TNF-α as well as HOMA-IR values. Ordinal logistic regression analysis revealed IP-10 was an independent risk factor associated with progressive liver injury, insulin resistance and incident diabetes. Circulating IP-10 may be a non-invasive biomarker for disease progression and subsequent diabetes development of NAFLD.

Nonalcoholic fatty liver disease (NAFLD) is a leading cause of chronic liver disease in Western societies^1^. In Asia, the community prevalence of NAFLD ranges from 15% to 45%, which is similar to that in Western countries^2^. The increasing trend in prevalence of NAFLD is mainly due to the pandemicity of obesity^3^. NAFLD includes a spectrum of clinical and pathological conditions that range from fatty liver alone to nonalcoholic steatohepatitis (NASH) with or without fibrosis^4^,^5^. Earlier work proposed that patients with NAFLD have a relatively benign liver prognosis^6^. However, recent studies have shown that NAFLD is associated with components of metabolic syndrome such as insulin resistance, abdominal obesity and a proinflammatory state, and increases the risk of developing type 2 diabetes mellitus^7^,^8^,^9^.

A growing body of evidence suggests that lipid gathering in hepatocytes triggers the production of proinflammatory factors, such as tumor necrosis factor alpha (TNF-α)^10^,^11^, interleukin-6 (IL6)^12^, and interferon gamma (IFN-γ)-induced protein 10 (IP-10)^13^. The active role of those cytokines might be crucial in the progression of NAFLD by inducing hepatic inflammation, hepatocyte apoptosis and necrosis, and fibrosis^11^,^12^,^14^.

IP-10, initially identified as an IFN-γ-inducible chemokine and functionally categorized as a proinflammatory chemokine, regulates immune responses by activating and recruiting leukocytes including T cells, eosinophils, monocytes and NK cells through binding to chemokine (C–X–C motif) receptor 3 (CXCR3)^15^. Moreover, IP-10 is secreted by a variety of cells including monocytes, neutrophils, endothelial cells, keratinocytes, fibroblasts, mesenchymal cells, dendritic cells, astrocytes and hepatocytes^15^. In the early 2000s, circulating IP-10 levels were shown to be increased in the early stage of type 1 diabetes^16^,^17^. More recent research has revealed that intrahepatic and circulating IP-10 is associated with obesity and insulin resistance in patients with chronic hepatitis C virus (HCV) infection and in patients with HCV/human immunodeficiency virus (HIV) co-infection^18^,^19^. Regarding IP-10 in type 2 diabetes, one previous population-based study demonstrated that IP-10 was neither significantly elevated in type 2 diabetes patients compared with control subjects nor associated with type 2 diabetes^20^. However, other studies have reported that levels of circulating IP-10 are increased in patients with type 2 diabetes^21^,^22^. Therefore, future studies are needed to address the clinical significance of circulating IP-10 in pre-diabetic and type 2 diabetic patients.

At present, there are no clear clinical guidelines to predict the progressive stages of NAFLD^24^. Liver biopsy is the gold-standard for direct diagnosis of NAFLD and evaluation of its progression; however, its use is only appropriate for selected individuals because of its invasiveness and cost^23^. Many non-invasive methods for diagnosing and staging NALFD as well as predicting its prognosis have been investigated, but none have been shown to be as accurate as liver biopsy^24^,^25^. Several studies have demonstrated that IP-10 plays a role in the development of intrahepatic inflammation and β cell degeneration^26^,^27^,^28^,^29^. More recently, IP-10 has been demonstrated to be a key player in the pathogenesis of experimental non-alcoholic steatohepatitis (NASH)^13^. However, few studies have examined the relationship between IP-10, components of metabolic syndrome and type 2 diabetes in patients with NAFLD. We hypothesized that circulating IP-10 plays an important role in insulin resistance and the development of type 2 diabetes in patients with NAFLD. To test our hypothesis, we measured differences in metabolic parameters, proinflammatory cytokines, endotoxin and biologic markers of oxidative stress in participants categorized according to an ordered outcome of NAFLD progression in humans^8^,^30^, namely participants without NAFLD, patients with NAFLD alone, and patients with NAFLD and incident diabetes.

## Results

### Demographic, Clinical and Laboratory Data

**Figure 1:**
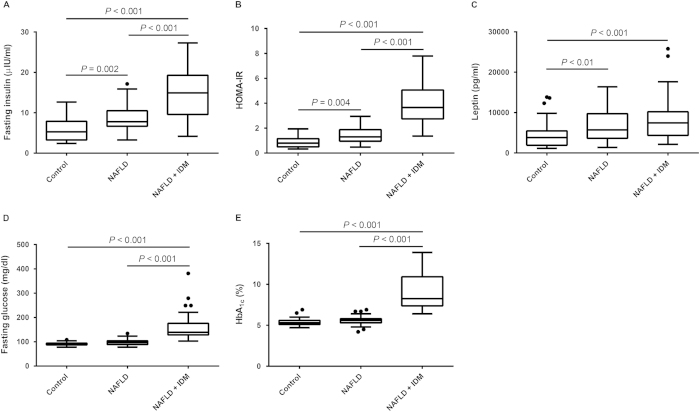
Clinical characteristics among the three groups. (**A**) Fasting insulin level, (**B**) HOMA-IR value and (**C**) leptin concentration were significantly higher in patients with NAFLD alone and in NAFLD patients with incident diabetes than in subjects without NAFLD (control group). (**A**) Fasting insulin, (**B**) HOMA-IR, (**D**) fasting glucose and (**E**) HbA_1c_ were significantly higher in subjects with NAFLD and incident diabetes than in subjects in the control and NAFLD alone groups. (**A**, **B**) An escalating trend in fasting insulin and HOMA-IR was observed among the three groups. (**A** - **E**) Box-and-whisker plots. The top and bottom of each box indicate the 1^st^ and 3^rd^ quartiles (interquartile range, IQR), and the band inside the box is the median. The ends of the whiskers represent the minima and maxima without outliers. The dot indicates an outlier more than 1.5 times the IQR below the 25^th^ percentile, or more than 1.5 times the IOR above the 75^th^ percentile of all of the data.

**Figure 2:**
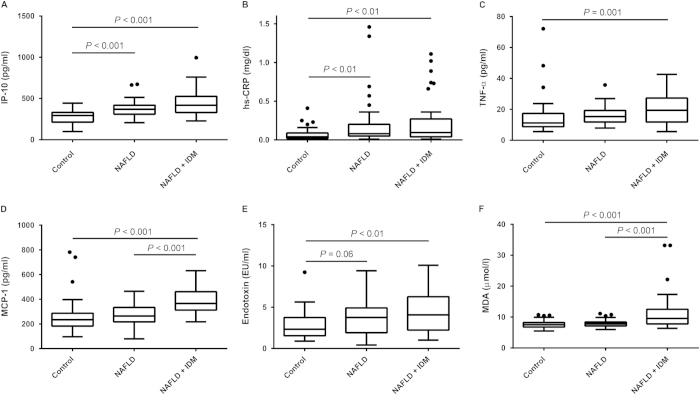
Circulating high-sensitivity CRP, proinflammatory cytokines, endotoxin and oxidative stress in the three groups. (**A**) IP-10 and (**B**) hs-CRP levels were significantly higher in patients with NAFLD alone and in NAFLD patients with incident diabetes than in subjects in the control group. (**C**) TNF-α and (**E**) endotoxin levels were significantly higher in patients with NAFLD and incident diabetes than in subjects in the control group. (**D**) MCP-1 and (**F**) MDA levels were significantly higher in NAFLD patients with incident diabetes than in patients with NAFLD alone and in control subjects. (**E**) An increasing trend in endotoxin was observed in patients with NAFLD alone.

Table 1 shows the demographic, clinical and laboratory data for the two groups of patients with nonalcoholic fatty liver disease (NAFLD alone, n = 48; NAFLD with incident diabetes, n = 42) and for control subjects (n = 43). The prevalence of overweight or obesity was 37.2% in the control group, 79.2% in the NAFLD group and 85.7% among NAFLD patients with incident diabetes (chi-square for trend *P* < 0.001). Patients with NAFLD and incident diabetes were more likely to be older, have higher systolic blood pressure (SBP), diastolic blood pressure (DBP), body mass index (BMI) and homeostasis model assessment of insulin resistance (HOMA-IR) values, higher serum aspartate aminotransferase (AST), alanine aminotransferase (ALT), fasting glucose, glycated hemoglobin (HbA_1c_), triglyceride, leptin, fasting insulin, high-sensitivity C-reactive protein (hs-CRP), plasma endotoxin, IP-10, monocyte chemoattractant protein-1 (MCP-1), TNF-α, and malondialdehyde (MDA) levels and lower serum high-density lipoprotein cholesterol (HDL-C) levels (Table 1, Figs. 1,2). Plasma IP-10 levels were significantly higher in patients with NAFLD alone (median (interquartile range): 369.44 (309.30–418.97) pg/mL) and in those with incident diabetes (418.99 (330.73–526.04) pg/mL) than in control subjects (293.37 (214.10–331.57) pg/mL) (*P* < 0.001) (Fig. 2A). Patients with NAFLD alone had higher SBP, DBP, BMI, and HOMA-IR values, higher serum ALT, triglyceride, fasting insulin, leptin, hs-CRP, and IP-10 levels, and lower HDL-C level than control subjects. Patients with NAFLD and incident diabetes had higher HOMA-IR values and higher fasting glucose, insulin, HbA_1c_, MCP-1 and MDA levels than patients with NAFLD alone and control subjects (Table 1, Figs. 1,2). In addition, plasma fasting insulin levels and HOMA-IR values significantly increased in a stepwise fashion from control subjects to patients with NAFLD alone to patients with NAFLD and incident diabetes (Fig. 1A,B).

### Circulating IP-10 and liver enzyme levels

**Figure 3:**
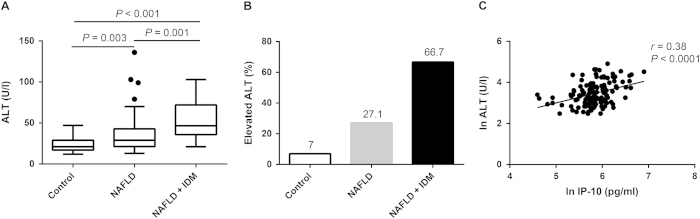
The relationship between liver function tests and circulating IP-10. (**A**) An escalating trend in serum ALT levels was observed among the three groups. (**B**) There were significant differences in proportions of participants with elevated liver enzymes among the three groups (*P* < 0.001 by chi-square test for trend). (**C**) Plasma IP-10 level was positively correlated with serum ALT in all participants.

Serum ALT significantly increased in a stepwise fashion from control subjects (21 [17–29] U/L), to patients with NAFLD (29 [21.3–42.8] U/L) to patients with NAFLD and incident diabetes (46.5 [35.8–72] U/L) (Fig. 3A). An increasing trend in prevalence of liver injury (elevated ALT) was observed among the three groups (chi-square for trend *P* < 0.001) (Fig. 3B). Additionally, plasma IP-10 levels were significantly correlated with serum ALT levels in all subjects (Fig. 3C).

### Correlations between circulating IP-10 and metabolic parameters

**Figure 4:**
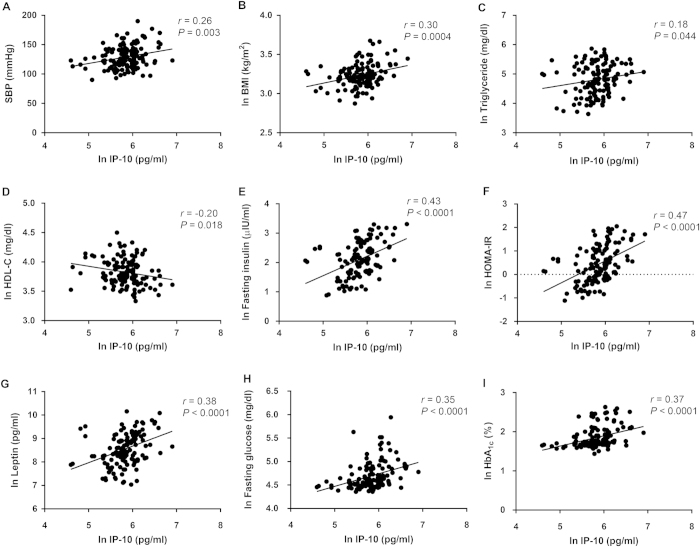
Circulating IP-10 correlates with metabolic syndrome. Univariate analysis of the correlation of plasma IP-10 level with (**A**) systolic blood pressure, (**B**) body mass index, (**C**) triglyceride, (**D**) HDL-C, (**E**) fasting insulin, (**F**) HOMA-IR, (**G**) leptin, (**H**) fasting glucose, and (**I**) HbA_1c_. The plasma or serum levels of IP-10, triglyceride, HDL-C, fasting insulin, HOMA-IR, leptin, fasting glucose, HbA1c and BMI were expressed as the natural logarithm (ln).

Plasma IP-10 was positively correlated with SBP (*r* = 0.26, *P* = 0.003), DBP (*r* = 0.18, *P* = 0.036), BMI (*r* = 0.30, *P* = 0.0004), triglyceride (*r* = 0.18, *P* = 0.044), fasting insulin (*r* = 0.43, *P* < 0.0001), HOMA-IR (*r* = 0.47, *P* < 0.0001), leptin (*r* = 0.38, *P* < 0.0001), fasting glucose (*r* = 0.35, *P* < 0.0001) and HbA_1c_ (*r* = 0.37, *P* < 0.0001) levels and was negatively correlated with HDL (*r* = −0.20, *P* = 0.018) (Fig. 4).

### Correlations between circulating IP-10, inflammation and oxidative stress

**Figure 5:**
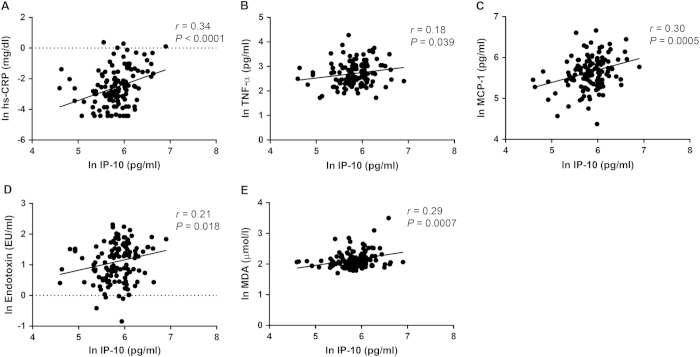
Circulating IP-10 correlates with inflammation marker, proinflammatory cytokines, endotoxinemia and oxidative stress. Positive associations between circulating IP-10 and (**A**) hs-CRP, (**B**) TNF-α, (**C**) MCP-1, (**D**) endotoxin and (**E**) MDA.

Plasma IP-10 level was positively correlated with plasma hs-CRP (*r* = 0.34, *P* < 0.0001), TNF-α (*r* = 0.18, *P* = 0.039), MCP-1 (*r* = 0.30, *P* = 0.0005), endotoxin (*r* = 0.21, *P* = 0.018) and MDA (*r* = 0.29, *P* = 0.0007) levels (Fig. 5).

### Multivariate ordinal logistic regression analyses to evaluate the association with progression of NAFLD, insulin resistance and incident diabetes

In the present study, liver injury was defined in patients with serum ALT levels above the upper limit (40 U/L) that could not be attributed to other medical causes (e.g. viral hepatitis). Insulin resistance was estimated by HOMA-IR, and incident diabetes was defined in patients with a fasting plasma glucose concentration ≥7.0 mmol/L. Table 2 shows the univariate and multivariate-adjusted odds ratios (ORs) for the progression of NAFLD, insulin resistance and incident diabetes associated with a unit increase of ln IP-10. IP-10 significantly predicted progressive liver disease, insulin resistance and incident diabetes with an OR of 31.02 [95% confidence interval (CI) 9.15–105.18] per 1 ln IP-10 unit of change. After adjustment for age and sex, traditional risk factors such as SBP, BMI, fasting glucose and HOMA-IR, and nontraditional possible confounders such as ALT, endotoxin, hs-CRP, leptin and MDA, the association between IP-10 and disease progression and incident diabetes remained significant (OR 7.84 [95% CI 1.51–40.82]).

### Circulating IP-10 is a potential diagnostic biomarker for NAFLD and incident diabetes

**Figure 6:**
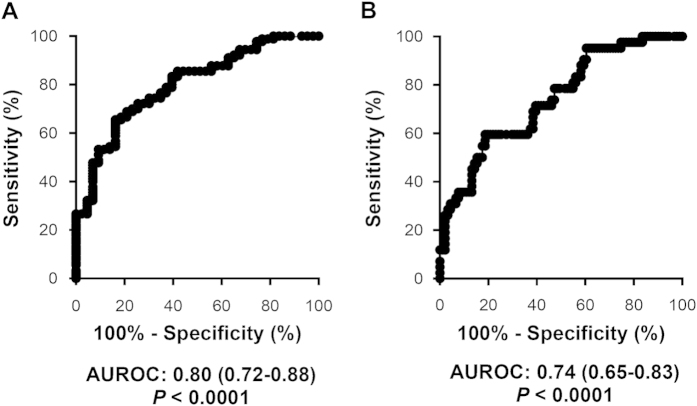
Values of IP-10 for the diagnosis of NAFLD and incident diabetes mellitus. Receiver-operating characteristic curves of plasma IP-10 in diagnosing (**A**) NAFLD and (**B**) incident diabetes in all subjects.

Receiver-operating characteristic (ROC) curves were constructed to evaluate whether IP-10 could serve as a diagnostic biomarker of NAFLD and incident diabetes. Plasma IP-10 exhibited a high accuracy in discriminating NAFLD from control subjects, with an area under the ROC curve (AUROC) of 0.80 (95% CI 0.72–0.88, *P* < 0.0001) (Fig. 6A). The AUROC for diagnosing incident diabetes was 0.74 (95% CI 0.65–0.83, *P* < 0.0001) (Fig. 6B).

## Discussion

In the present study, we examined the relationship between IP-10 and progression from non-NAFLD to NAFLD to NAFLD with incident diabetes. We found that circulating plasma IP-10 levels were significantly higher in patients with NAFLD alone (*P* < 0.001) and in patients with NAFLD and incident diabetes (*P* < 0.001) than in controls. We also found that IP-10 levels were positively correlated with ALT, HOMA-IR, hs-CRP, MDA, MCP-1, and TNF-α. Further analysis revealed that IP-10 was an independent risk factor associated with progressive liver injury, insulin resistance and incident diabetes in NAFLD patients.

A few studies have suggested that IP-10 is associated with the development of intrahepatic inflammation, obesity and diabetes. For instance, Bertola *et al.* found that the IP-10 gene was significantly upregulated in liver of obese patients with NASH^28^. Schulthess *et al.* found that IP-10 impairs β cell function via TLR4 signaling in patients with type 2 diabetes^29^, and Morimoto *et al.* demonstrated that IP-10 neutralization enhanced β cell proliferation and suppressed diabetes occurrence in non-obese diabetic mice^31^. In an elegant experiment involving Cxcl10 gene knockout (Cxcl10^-/-^) mice, Zhang and colleagues recently found that IP-10 was an independent risk factor for the development of NASH^13^. Although these studies provide a better understanding of the role IP-10 plays in the development of NAFLD and its link to β cell destruction in diabetes, they provide limited information about the association between IP-10 and metabolic components. Our study is the first to investigate the association between IP-10, insulin resistance, and the development of type 2 diabetes simultaneously in people with NAFLD. We found that plasma IP-10 levels were significantly higher in patients with NAFLD alone and in those with incident diabetes than in healthy individuals. Ordinal logistic regression analysis further revealed that IP-10 was closely associated with progressive liver injury, insulin resistance and incident diabetes. The results are consistent with those in previous studies in some respects. However, previous studies did not investigate the relationship between IP-10 and type 2 diabetes development in humans. We believe our results increase the reliability of the association between IP-10 and progressive NAFLD; however, a longitudinal study is needed to confirm the role IP-10 plays in the development of NAFLD and incident diabetes.

Leptin, an adipocyte-derived hormone, has been demonstrated to directly or indirectly affect insulin sensitivity through modulation of insulin signaling and the molecules involved in glucose and lipid metabolism^32^. High leptin concentrations may contribute to insulin resistance^32^ and serve as a potent predictor of the development of insulin resistance and its related cardiovascular risk^33^. A few studies have investigated the relationship between IP-10 and leptin. Meier *et al.* found that leptin could induce the expression and secretion of IP-10 in a human monocytic cell line and in peripheral blood mononuclear cells^34^, and Jain *et al.* reported that plasma IP-10 levels were positively associated with plasma levels of leptin in type 2 diabetic patients^21^. Another study reported that increased serum levels of leptin were related to those of IP-10 in normal pregnancy and in preeclampsia^35^. To the best of our knowledge, our study is the first to report that plasma IP-10 and leptin levels are significantly higher in patients with NAFLD alone and in those with incident diabetes. Moreover, we found that circulating IP-10 levels were closely correlated with serum leptin concentration and metabolic parameters (blood pressure, BMI, triglyceride, HDL-C, HOMA-IR, fasting glucose and HbA_1c_). Further studies are needed to confirm whether IP-10 collaborates with leptin in the pathogenesis of NAFLD, insulin resistance and the development of incident diabetes.

Endotoxin, a key component of many bacterial species present in the human microbiota, plays a central role in innate immune responses and has been considered a factor that influences energy absorption, systemic inflammation, and the development of insulin resistance^36^. A few studies have shown that increased levels of circulating endotoxin are associated with insulin resistance in NAFLD patients^37^,^38^. Consistently, our results demonstrated a significant upward trend in plasma endotoxin levels among the three groups. Additionally, we found a significant association between circulating endotoxin and IP-10 levels in our three groups of participants. This positive correlation may indicate that endotoxin and IP-10 simultaneously play a key role in the pathogenesis of NAFLD, insulin resistance and incident diabetes.

We found that NAFLD is closely associated with components of metabolic syndrome (Figs. 1 and 4), indicating that the disease might be a hepatic manifestation of that syndrome. Kotronen *et al.* reported similar findings^7^. Studies have shown that NAFLD is an independent risk factor for subsequent development of type 2 diabetes^9^ and that patients with NAFLD and diabetes are at increased risk of a poor outcome, such as cirrhosis development and death^39^. In the current study, we found that increased plasma IP-10 level was associated with progression of NAFLD and incident diabetes. We also measured other pathogenic factors that have been shown to be related to the development of NAFLD or diabetes^33^,^37^,^38^,^40^,^41^, such as TNF-α, MCP-1, leptin, and endotoxin (Figs. 1 and 2) as well as MDA, a marker of oxidative stress (Fig. 2). When standardized in IP-10 and these factors, analysis revealed that IP-10 was a very strong and independent factor associated with outcome of NAFLD progression (OR 3.05 [95% CI 1.86–5.00], *P* < 0.0001) (Supplementary Table 1). In addition, ROC curve analysis further revealed that plasma IP-10 had a fair to good diagnostic accuracy (AUROC = 0.74, *P* < 0.0001). Although NAFLD is believed to be a strong determinant for the development of metabolic syndrome and cardiovascular diseases, there is currently no established standard to identify NAFLD patients at risk of disease progression. Our results suggest that IP-10 could serve as a potential indicator of NAFLD development alone or NAFLD with incidental type 2 diabetes.

There are several limitations to our study. First, we used a cross-sectional design rather than a longitudinal design with a relatively small sample size. The cross-sectional design does not allow us to infer causal relationships and the small sample size may reduce the statistical power of this study. Further longitudinal studies with a larger sample size may be beneficial to establish the cause–effect relationships. Second, the diagnosis of NAFLD was based on magnetic resonance imaging (MRI) findings instead of liver biopsy, the gold standard for diagnosis and staging of NAFLD. Liver biopsy is invasive, expensive, and thus impractical for routine clinical use in all NAFLD patients^23^. Third, alcohol intake, another common cause of fatty liver, was self-reported. Patients’ self-reporting of alcohol intake may lead to an underestimation of alcoholic fatty liver^9^. However, none of our participants had serum gamma glutamyl transferase levels above 100 U/L, a well-known biomarker for alcohol consumption. Finally, HOMA-IR may not have been the best index for assessing insulin resistance. More accurate assessments can be made by more accomplished measurements, such as the hyperinsulinemic-euglycemic clamp and the intravenous glucose tolerance test.

In summary, we found that circulating IP-10 level was closely associated with insulin resistance, endotoxemia, oxidative stress and increased levels of inflammatory cytokines, which are characteristics of the development and progression of NAFLD. Among the potential pathogenic factors examined in this study, IP-10 was a very strong and independent factor related to the development of liver injury, insulin resistance and incident diabetes in patients with NAFLD. Our results suggest that circulating IP-10 is a potential non-invasive indicator of NAFLD progression. However, our cross-sectional study had a limited capability for causal inference. Further longitudinal studies are required to clarify whether IP-10 is a clinically useful biomarker for the development of type 2 diabetes and a therapeutic target in patients with NAFLD.

## Methods

### Study design and participants

This cross-sectional, case-control study comprising 133 adult participants was conducted at the Changhua Christian Hospital Health Examination Center during the period February 1, 2012 to January 31, 2014. The study was carried out in strict accordance with guidelines for research involving human subjects developed by the Taiwan Ministry of Health and Welfare. All experimental protocols were approved by the Institutional Review Board of the Changhua Christian Hospital (approval numbers 110507 and 110601). None of the participants had a history of systemic disease, alcoholism or HBV, HCV or HIV infection. Also, none of the participants were being treated with insulin injections, oral antidiabetic agents, antioxidants such as vitamins C or E, immunosuppressants, non-steroidal anti-inflammatory or hepatotoxic drugs. All of the participants provided written informed consent to participate in the study before undergoing MRI to test for the presence of NAFLD and before blood tests were performed to measure metabolic syndrome-associated profiles as well as endotoxin, proinflammatory cytokines and oxidative stress marker (MDA) levels. All participants visited the Health Examination Center after an overnight fast for anthropometric measurements and blood tests. The diagnosis of diabetes mellitus was made according to American Diabetes Association criteria^42^.

### Magnetic resonance imaging for NAFLD

Subjects were examined in the supine position with a standard four-channel torso phased-array coil centered over the liver at 1.5 Tesla (Siemens Symphony, Siemens Medical Systems, Erlangen, Germany). Images were acquired in the axial plane during an end-expiratory breath-hold using a sensitivity encoding technique. The two-point Dixon method as modified by Fishbein was used to measure the fraction of hepatic fat^43^. Briefly, the method is based on phase-shift imaging in which hepatic fat fraction (HFF) is calculated from the signal difference between the vectors resulting from in-phase (IP) and out-of-phase (OP) signals. Hepatic fatty infiltration was assessed on breath-hold T1-weighted dual gradient-echo sequences. The sequence parameters included a repetition time of 90-200 milliseconds (msec), an echo time of 2.1 msec for OP images and 4.2 msec for IP images; flip angle, 70°; section thickness, 8 mm; matrix size, 256 × 128-192; field of view, 32 cm × 40 cm. Pixel signal intensities from IP and OP images were obtained from three selected regions of interest. Spleen was also measured at the corresponding liver levels to adjust for the lack of an objective signal intensity scale 44. Liver fat fraction was measured from magnetic resonance images using the formula: FFMRI = (S_in_-S_out_)/(2S_in_), in which FFMRI = fat fraction on MRI, S = an average signal intensity of liver divided by spleen, S_in_ = signal intensity on IP images, and S_out_ = signal intensity on OP images. MRI results were analyzed by an experienced radiologist (C.-T. C., with 8 years of experience) who was blinded to the clinical and laboratory findings.

### Laboratory Methods

Serum AST, ALT, total cholesterol, triglyceride, low-density lipoprotein cholesterol (LDL-C), HDL-C, fasting glucose, insulin, HbA_1c_, hs-CRP and leptin were measured using standardized procedures at the Department of Laboratory Medicine, Changhua Christian Hospital. Insulin resistance was assessed based on the homeostasis model assessment of insulin resistance (HOMA-IR) using the following formula: (fasting glucose × fasting insulin)/22.5 (fasting glucose was measured in millimoles per liter and fasting insulin in milliunits per liter)^45^. Plasma endotoxin, IP-10, MCP-1, TNF-α and MDA were measured using specific methods at the Inflammation Research and Drug Development Center, Changhua Christian Hospital, and are described as follows.

### Circulating endotoxin and cytokines

Plasma endotoxin levels were measured by a chromogenic Limulus Amebocyte Lysate assay (QCL-1000™; Lonza, Walkersville, MD, USA). Plasma levels of TNF-α were measured using a highly sensitive magnetic bead-based assay kit (MILLIPLEX MAP Kit, High Sensitivity Human Cytokine Magnetic Bead Kit – Premixed; EMD Millipore, Billerica, MA, USA). The levels of plasma IP-10 and MCP-1 were assessed using another magnetic bead-based kit (MILLIPLEX MAP Kit, Human Cytokine/Chemokine Magnetic Bead Panel; EMD Millipore). All assays were performed in accordance with the manufacturers’ instructions.

### Circulating malondialdehyde (MDA)

MDA is one of the most frequently used indicators of oxidative stress^46^. Plasma MDA was assayed with the Thiobarbituric Acid Reactive Substances (TBARS) Assay kit based on the manufacturer’s instructions (Cayman Chemical Company, Ann Arbor, MI, USA). Absorbance of the samples was measured at 535 nm using a VERSA brand microplate reader (designed by Molecular Devices, Sunnyvale, CA, USA). The concentration of MDA was determined using an MDA standard curve.

### Statistical analysis

Results are expressed as a percentage, median (interquartile range) or mean ± standard deviation. Each variable was tested for normal distribution using the Kolmogorov-Smirnov test. Non-normally distributed variables were either log-transformed (ln) for correlation and regression analysis or analyzed using nonparametric statistical tests. Multiple comparisons among the three groups were made using the ANOVA test with Bonferroni correction or the Kruskal–Wallis test followed by the Dunn test for continuous variables or the Pearson chi-squared test for categorical variables. Correlations between pairs of continuous variables were determined by the Pearson correlation test. Univariate ordinal logistic regression analysis was performed to assess the association between IP-10 and progression of the disease (liver injury and insulin resistance) and incident diabetes, followed by multivariate ordinal logistic regression analyses with regard to potential confounders. In the first model, multivariate ordinal regression analyses between IP-10 and progression of the disease with incident diabetes were adjusted for age and sex. In the second model, we adjusted for age and sex plus traditional risk factors such as SBP, BMI, fasting glucose, total cholesterol and HOMA-IR. In the full model, we kept age, sex and all of the traditional risk factors and additionally adjusted for nontraditional risk factors such as ALT, endotoxin, hs-CRP, leptin and MDA. All statistical analyses were performed using GraphPad Prism 5 (GraphPad Software, San Diego, CA, USA) and IBM SPSS 20 (SPSS, Inc., Chicago, IL, USA). In all analyses, *P*-values of <0.05 were considered statistically significant.

## Author Contributions

All authors reviewed the manuscript. C.-L.W. performed the analysis, and wrote the manuscript. H.-M.W. and C.-C.C. conceived and designed the experiments. C.-T.C., T.-Y.C. and H.-M.W. performed the experiments and collected the data. C.-L.W., H.-M.W., D.-C.T., C.-T.C., C.-T.K., K.-L.S., W.-W.S. and C.-C.C. contributed to the discussion and manuscript revision.

## Additional Information

**How to cite this article**: Chang, C.-C. *et al*. Interferon gamma-induced protein 10 is associated with insulin resistance and incident diabetes in patients with nonalcoholic fatty liver disease. *Sci. Rep.* **5**, 10096; doi: 10.1038/srep10096 (2015).

## Supplementary Material

Supplementary Information

## Figures and Tables

**Table 1:** Clinical characteristics and laboratory data of controls and patients with nonalcoholic fatty liver disease with and without incident diabetes mellitus.

	**Control**	**NAFLD**		***P*** **for trend**^a^
		**NAFLD alone**	**NAFLD with IDM**	
	**n=43**	**n=48**	**n=42**	
Age, years	53.0 (48.0–58.0)	53.5 (49.25–58.75)	57.0 (52.0–61.0)^b^	0.018
Male, n (%)	21 (48.8)	27 (56.3)	22 (52.4)	0.778
SBP, mm Hg	118.33±13.35	130.52±17.51^c^	137.83±20.95^d^	<0.001
DBP, mm Hg	77.0 (71.0–82.0)	84.0 (77.25–89.0)^c^	84.0 (79.0–90.0)^c^	<0.001
BMI, kg/m^2^	23.0 (20.80–24.90)	25.05 (24.15–27.68)^d^	26.71 (24.77–30.58)^d^	<0.001
AST, U/L	24 (22–30)	27 (21–34)	36.5 (29–52)^d^, ^e^	<0.001
ALT, U/L	21 (17–29)	29 (21.3–42.8)^c^	46.5 (35.8–72)^d^, ^e^	<0.001
Cholesterol, mg/dL	203.28±37.35	208.06±39.54	209.50±31.58	0.711
Triglyceride, mg/dL	83.0 (58.0–108.0)	146.0 (103.25–213.0)^d^	159.5 (119.25–213.75)^d^	<0.001
LDL-C, mg/dL	129.98±32.03	132.53±33.13	133.19±30.48	0.886
HDL-C, mg/dL	53.0 (45.0–62.0)	43.0 (39.25–48.75)^d^	41.0 (36.75–47.0)^d^	<0.001

All variables are expressed as n (%) for categorical data and as means ± SD or medians (interquartile ranges) for continuous data with or without a normal distribution, respectively. Abbreviations: ALT, alanine aminotransferase; AST, aspartate aminotransferase; DBP, diastolic blood pressure; HDL-C, high density lipoprotein cholesterol; IDM, incident diabetes mellitus; LDL-C, low density lipoprotein cholesterol; NAFLD, nonalcoholic fatty liver disease; SBP, systolic blood pressure.

^a^*P* value by ANOVA or the Kruskal–Wallis test for continuous variables and Pearson Chi-squared test for categorical variables.

^b^*P *< 0.05 vs. control.

^c^*P *< 0.01 vs. control.

^d^*P *< 0.001 vs. control.

^e^*P *< .01 vs. NAFLD alone.

**Table 2:** Multivariate-adjusted ordinal logistic regression analyses for odds ratios of progressive liver injury, insulin resistance and incident diabetes for plasma IP-10.

**Analysis**	**Progressive liver injury, insulin resistance, and incident diabetes**		
	**Odds ratio**	***P*** **Value**	**95% Confidence Interval**
Univariate			
ln IP-10, pg/ml	31.02	<0.001	9.15–105.18

Multivariate			
Model 1	32.62	<0.001	9.01–118.05
Model 2	9.90	0.005	1.98–49.58
Model 3	7.84	0.014	1.51–40.82

Model 1 is adjusted for IP-10 plus age and sex. Model 2 is adjusted for the variables in model 1 plus traditional risk factors (SBP, BMI, fasting glucose, total cholesterol and HOMA-IR); Model 3 is adjusted for the variables in model 2 plus nontraditional risk factors (ALT, endotoxin, hs-CRP, leptin and MDA). The Nagelkerke pseudo *R*^*2*^ of model 3 was 0.836. Logarithmic transformation (ln) of IP-10, BMI, fasting glucose, HOMA-IR, ALT, endotoxin, hs-CRP, leptin and MDA was used to normalize the distributions for multivariate analysis.
