# Supplementary material for: Interferon gamma-induced protein 10 is associated with insulin resistance and incident diabetes in patients with nonalcoholic fatty liver disease
Source: Sci Rep. 2015 May 11;5:10096. doi: 10.1038/srep10096 (PMC4426720; doi:10.1038/srep10096)
Supplement: Supplementary Information [file srep10096-s1.doc]

**Interferon gamma-induced protein 10 is associated with insulin resistance and incident diabetes in patients with nonalcoholic fatty liver disease**

Chia-Chu Chang^1,2†^, Chia-Lin Wu^1,2,3†^, Wei-Wen Su^4^, Kai-Lun Shih^4^, Der-Cherng Tarng^3,5,6^, Chen-Te Chou^7^, Ting-Yu Chen^8^, Chew-Teng Kor^9^ and Hung-Ming Wu^8,10,11*^

^1^ Division of Nephrology, Department of Internal Medicine, Changhua Christian Hospital, Changhua, Taiwan

^2^ School of Medicine, Chung-Shan Medical University, Taichung, Taiwan

^3^ Institute of Clinical Medicine, National Yang-Ming University, Taipei, Taiwan

^4^ Department of Gastroenterology, Changhua Christian Hospital, Changhua, Taiwan

^5^ Division of Nephrology, Department of Medicine, Taipei Veterans General Hospital, Taipei, Taiwan

^6^ Department and Institute of Physiology, National Yang-Ming University, Taipei, Taiwan

^7^ Department of Medical Imaging, Changhua Christian Hospital, Changhua, Taiwan

^8^ Inflammation Research & Drug Development Center, Changhua Christian Hospital, Changhua, Taiwan

^9^ Internal Medicine Research Center, Changhua Christian Hospital, Changhua, Taiwan

^10^ Graduate Institute of Acupuncture Science, China Medical University, Taichung, Taiwan

^11^ Department of Neurology, Changhua Christian Hospital, Changhua, Taiwan

^†^ These two authors contributed equally to this work.

^*^ ***Corresponding author and person to whom reprint requests should be addressed*:**

Hung-Ming Wu, MD, PHD

Inflammation Research & Drug Development Center

Changhua Christian Hospital

No. 135 Nan-Siau Street, Changhua 500, Taiwan

Tel: +886-4-7238595 ext. 4237

E-mail: [18288@cch.org.tw](mailto:18288@cch.org.tw)

Supplementary Table 1. Comparisons of the association between potential pathogenic factors (IP-10, MCP-1, TNF-α, leptin, endotoxin, and MDA) and outcome^a^ in multivariate ordinal logistic regression^b^

| Pathogenic factors | Coef. | S.E. | Wald | *P* value | OR^c^ | 95% CI |
| --- | --- | --- | --- | --- | --- | --- |
| Standardized^d,e^ ln IP-10 | 1.114 | 0.253 | 4.400 | <0.0001 | 3.047 | 1.855−5.003 |
| Standardized ln MDA | 1.006 | 0.279 | 3.600 | <0.0001 | 2.736 | 1.583−4.729 |
| Standardized ln MCP-1 | 0.878 | 0.230 | 3.810 | <0.0001 | 2.405 | 1.532−3.776 |
| Standardized ln Endotoxin | 0.444 | 0.194 | 2.300 | 0.022 | 1.559 | 1.067−2.278 |
| Standardized ln TNF-α |  |  |  | NS |  |  |
| Standardized ln Leptin |  |  |  | NS |  |  |
| Interception for NAFLD | 1.350 | 0.257 | 5.240 | <0.0001 |  |  |
| Interception for NAFLD and incident diabetes | -1.167 | 0.252 | -4.630 | <0.0001 |  |  |

^a^ Progressive fatty liver disease, insulin resistance and incident diabetes

^b^ Backward stepwise elimination was used. *R^2^* of this model was 0.545.

^c^ Odds ratio (per 1 SD in ln pathogenic factor)

^d^ Standardized ln pathogenic factor = (ln pathogenic factor - mean(ln pathogenic factor))/SD(ln pathogenic factor)

^e^ Natural log-transformed pathogenic factor distributions were standardized to a mean of zero and standard deviation (SD) of 1 to facilitate comparison of effects sizes between pathogenic factors.

Abbreviations: Coef., standardized coefficients; NS, not significant; S.E., standard error.
